# Nitric Oxide Induced *stx2* Expression Is Inhibited by the Nitric Oxide Reductase, NorV, in a Clade 8 *Escherichia coli* O157:H7 Outbreak Strain

**DOI:** 10.3390/microorganisms10010106

**Published:** 2022-01-05

**Authors:** Rim Al Safadi, Michelle L. Korir, Shannon D. Manning

**Affiliations:** Department of Microbiology and Molecular Genetics, Michigan State University, East Lansing, MI 48824, USA; rimalsafadi@gmail.com (R.A.S.); mkorir@aurora.edu (M.L.K.)

**Keywords:** Shiga toxin, *Escherichia coli*, O157, virulence, pathogenesis, nitric oxide

## Abstract

*Escherichia coli* O157:H7 pathogenesis is due to Shiga toxin (Stx) production, though variation in virulence has been observed. Clade 8 strains, for instance, were shown to overproduce Stx and were more common among hemolytic uremic syndrome cases. One candidate gene, *norV*, which encodes a nitric oxide (NO) reductase found in a clade 8 O157:H7 outbreak strain (TW14359), was thought to impact virulence. Hence, we screened for *norV* in 303 O157 isolates representing multiple clades, examined *stx2* expression following NO exposure in TW14359 for comparison to an isogenic mutant (Δ*norV*), and evaluated survival in THP-1 derived macrophages. *norV* was intact in strains representing clades 6–9, whereas a 204 bp deletion was found in clades 2 and 3. During anaerobic growth, NO induced *stx2* expression in TW14359. A similar increase in *stx2* expression was observed for the Δ*norV* mutant in anaerobiosis, though it was not impaired in its ability to survive within macrophages relative to TW14359. Altogether, these data suggest that NO enhances virulence by inducing Stx2 production in TW14359, and that toxin production is inhibited by NorV encoded by a gene found in most clade 8 strains. The mechanism linked to these responses, however, remains unclear and likely varies across genotypes.

## 1. Introduction

Shiga toxin-producing *Escherichia coli* (STEC) O157:H7 is a leading cause of gastrointestinal illness linked to food and waterborne outbreaks worldwide. Patients infected with STEC O157 often present with hemorrhagic colitis, although some can develop hemolytic uremic syndrome (HUS) [1] that can lead to kidney failure and death. In the U.S., an annual 46.2% hospitalization rate and 0.5% death rate were reportedly attributable to STEC O157 [2], while the incidence of all STEC infections was estimated to be 5.9 cases per 100,000 individuals in 2018 [3]. Considerable variation in the number of infections, however, has been documented across different regions in the U.S. [4]

STEC pathogenesis is mainly due to the production of one or more Shiga toxins (Stx) encoded by genes carried on lambda-like bacteriophages [5]. Other virulence factors are also important. For instance, STEC strains possessing genes like *eae* (intimin) found on the locus of enterocyte effacement (LEE) pathogenicity island [6], contribute to attaching and effacing lesion formation on intestinal epithelial cells. *E. coli* strains possessing *stx* as well as the LEE island are classified as enterohemorrhagic *E. coli*. (EHEC), which were found to be more virulent than STEC strains lacking these factors [7]. Furthermore, EHEC strains producing the Stx2a and/or Stx2c variants were observed to be more virulent causing a higher frequency of HUS than strains producing the Stx1 variant alone or with Stx2a [5,8,9]. Differences have been reported across populations, however, indicating that strain specific factors are also important for clinical phenotypes. Indeed, we previously showed that a diverse set of O157 strains could be classified into nine distinct clades based on the phylogenetic analysis of 96 single nucleotide polymorphisms (SNPs) [10]. Most importantly, strains belonging to clade 8 were significantly more common among HUS cases and more frequently possessed genes encoding Stx2a and/or Stx2c compared to other clades. Follow up studies showed that clade 8 strains also had greater *stx2* expression levels following exposure to epithelial cells [11] and more than the expected number of Stx-encoding bacteriophage insertion sites occupied by prophages lacking *stx* [12]. Notably, deletion of a non-Stx-encoding prophage in a high *stx2a*-expressing clade 8 strain (TW14313) drastically reduced *stx2a* expression levels in the mutant relative to the wild type [12]. Taken together, these data suggest that clade 8 strains possess unique traits that may enhance their ability to cause more severe disease.

A genome analysis of the clade 8 O157:H7 strain (TW14359), which was implicated in the 2006 North American spinach outbreak that caused a higher-than-average frequency of HUS [13], uncovered several traits that could promote virulence [14]. One candidate gene, *norV*, encodes a nitric oxide (NO) reductase (NorV) that detoxifies NO in oxygen limiting conditions [15]. Two versions of *norV* have been identified [14]. An intact functional *norV* was detected in TW14359 [14], while a non-functional copy containing a 204 bp deletion was found in the EDL933 (clade 3) [16] and the Sakai (clade 1) [17] O157:H7 outbreak strain genomes [14]. An initial screen of 100 O157:H7 strains recovered from multiple sources detected the 204 bp deletion in 58% of strains and a correlation with *stx1* [14], which we found to be overrepresented among strains representing clades 1–3 [10]. These findings indicate that *norV* functionality and its impact on virulence may be restricted to strains with a specific genetic background.

A prior study also demonstrated that possession of an intact *norV* protected different EHEC O157:H7 strains from growth inhibition by NO under anaerobic conditions unlike strains possessing the non-functional version of *norV* [18]. Additionally, mutants lacking *norV* had decreased survival within macrophages. Since macrophage uptake and the subsequent production of NO are a critical part of the innate immune response to bacterial pathogens, these data suggest that possession of a functional *norV* can protect a bacterium from macrophage killing. Indeed, the same study found that insertion of a functional *norV* into the EDL933 chromosome subsequently increased *stx2* expression in the presence of NO inside macrophages [18]. As these data were generated with a strain that was not classified as clade 8, the function of *norV* in a more virulent strain background is not known. Consequently, the goal of this study was to examine the effects of NO and *norV* mutagenesis on growth, *stx2* expression, and macrophage survival using the clade 8 spinach outbreak strain, TW14359.

## 2. Materials and Methods

### 2.1. Bacterial Strains, norV Detection, and Growth Conditions

The *E. coli* O157:H7 spinach outbreak strain, TW14359, was used for all experiments; it possesses *stx2a* and *stx2c* and was previously classified as clade 8 by SNP genotyping [10]. This strain was previously recovered by the Michigan Department of Health and Human Services (MDHHS) from a patient who had consumed spinach linked to the national 2006 outbreak [13]. An additional set of 303 previously characterized O157 strains with seven *stx* profiles representing the predominant clades 1–3 and 6–9 [10], was screened for the presence of an intact (functional) or deleted version of *norV*. These strains were recovered from clinical cases in Michigan between 2001 and 2006 as described [19] Differences in the frequencies of *stx* profiles and *norV* type were detected using the likelihood chi-square test or Fisher’s exact test for sample sizes less than five.

After overnight growth in Luria–Bertani (LB) broth at 37 °C and DNA isolation, PCR was performed to detect the presence of a functional *norV* in all 303 strains. The primer set norV698_F and norV1373_R, which targets the region flanking the *norV* deletion (Table 1), was used with the following PCR conditions: 10 min at 94 °C plus 30 cycles of 1 min at 92 °C, 30 s at 53 °C and 1 min at 72 °C followed by 20 min at 72 °C.

### 2.2. Construction of a norV Mutant

*norV* was deleted from the chromosome of wild-type (WT) strain TW14359 using the red recombination procedure [20]. Briefly, recombinant PCR products containing a kanamycin (Km) resistance marker flanked by 50 bp sequences homologous to the upstream and downstream regions of *norV* were generated from plasmid pKD4 [21,22] using primers norv-del1/del2 (Table 1). PCR products were electroporated into the red recombinase-producing WT strain containing pKM208 as described previously [23]. Transformants were identified by growth on LB agar supplemented with 25 μg/mL Km following incubation at 37 °C. The deletion in *norV* (Δ*norV*) was confirmed by PCR-based restriction fragment length polymorphism using the norv-del3/del4 primer set followed by digestion with *Pst*I for 2 h at 37 °C.

For complementation of Δ*norV*, a 1620 bp region containing the *norV* coding region plus additional flanking regions including the *norV* promoter, were amplified by PCR from strain TW14539 genomic DNA using *TaKaRa LA Taq* polymerase (Takara Bio; Madison, WI, USA) with the norv-compl_F/norvcompl_R primer set. The resulting PCR products were cloned into the pCR2.1-TOPO vector (Invitrogen; Carlsbad, CA, USA) to construct the pCR2.1-TOPO*norV* plasmid. This plasmid was transformed into the Δ*norV* strain, creating the Δ*norV::norV*_WT_ strain. Next, we electroporated the pCR2.1-TOPO empty vector into the Δ*norV* strain, which resulted in a Δ*norV*::*vector* strain for use as a control. Transformants were grown overnight in LB supplemented with 100 μg/mL ampicillin at 37 °C.

### 2.3. Bacterial Gene Expression

Prior to RNA extraction, the WT and mutant strains were grown for 6 and 24 h in LB broth at 37 °C in the presence or absence of 200 μM of the nitric oxide donor (NOR4) (Sigma-Aldrich; Burlington, MA, USA). The concentration of NO was determined using the Nitrate/nitrite Colorimetric Assay Kit (Sigma-Aldrich). Strains were cultivated at 37 °C with shaking (aerobic) or without shaking in the presence of 5% CO_2_ (anaerobic). cDNA was synthesized from the extracted RNA by reverse transcription as described previously [24]; three independent RNA populations were extracted per sample. Quantitative real-time PCR (qRT-PCR) was performed utilizing amplification conditions and primers specific for the B subunit gene of *stx2*, that differentiates between *stx2a* and *stx2c* as was carried out previously [11,24]. Expression data are reported for “*stx2*” for simplicity. For normalization, published conditions and primers were used to amplify the 16S rRNA gene, *rrsH* [24]. Transcription differences were quantified as described [11,22], and expression levels were presented for strain TW14359 relative to Δ*norV* or the same strain grown in different conditions (e.g., aerobic vs. anaerobic). Fold change in *stx2* expression was calculated using the ddCt method [25]; a fold change of ≥2 was considered biologically significant.

### 2.4. Cell Culture and EHEC Infection

THP-1 monocyte-like cells (ATCC TIB-202) were cultured in Roswell Park Memorial Institute 1640 (RPMI) growth medium (Gibco; Amarillo, TX, USA) supplemented with 2 mM L-glutamine (Gibco), 10% fetal bovine serum (FBS; Atlanta Biologicals; Minneapolis, MN, USA), and 1% penicillin/streptomycin (Gibco) at 37 °C with 5% CO_2_. Cells were differentiated into macrophages by incubation with 100 nM phorbol 12-myristate 13-acetate (PMA; Sigma-Aldrich, St. Louis, MO, USA) in RPMI medium with 2% FBS for 24 h as was described [26]. Using a published protocol [27] that was modified for EHEC O157:H7, the cells were seeded at a density of 1 × 10^6^ cells per well in 24 well plates. Bacteria were added to the cell monolayer at a multiplicity of infection (MOI) of 10 and incubated at 37 °C with 5% CO_2_ for 1 h. The cells were washed with phosphate-buffered saline (PBS) and fresh medium containing 100 mg/mL of gentamicin was added for 1 h to kill extracellular bacteria. The infected monolayers were lysed by adding 0.1% triton X-100 (Sigma-Aldrich) in PBS. Lysates were diluted and plated onto LB agar and incubated at 37 °C overnight to quantify colony forming units (CFUs). The number of intracellular (phagocytosed) bacteria was determined by normalizing to the total number of bacteria left in the well after the 1 h infection period. The percent survival was calculated by dividing the number of intracellular bacteria at 24 h by the total number of intracellular bacteria 1 h after adding antibiotics and multiplying by 100. A Student’s t-test was used to determine if there were significant differences between strains as measured using the mean; a *p*-value ≤ 0.05 was considered significant.

## 3. Results

### 3.1. norV Distribution by Clade and Association with Stx Gene Profiles

**Figure 1:**
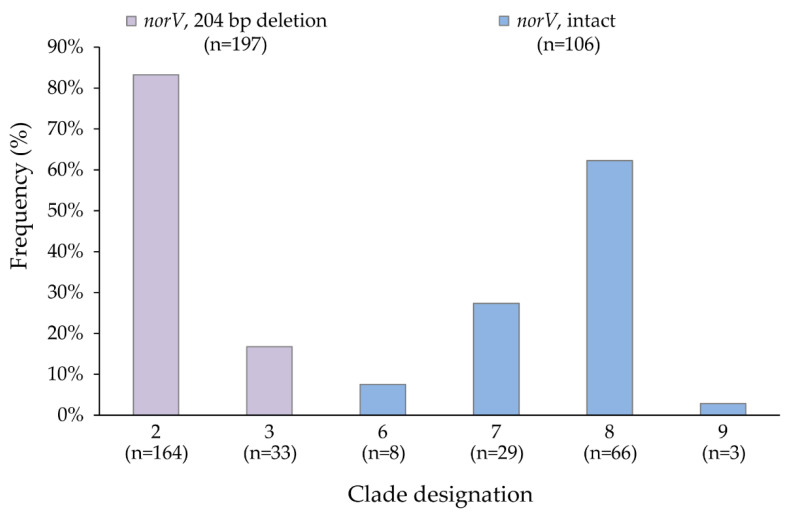
Distribution and percent of *norV* variants among 303 Shiga toxin-producing *Escherichia coli* O157 clinical isolates. Percentages for clades 2 and 3 were calculated using the total number of isolates with a deletion in *norV* (*n* = 197, purple bars) as the denominator, whereas clades 6–9 used the total number with an intact *norV* (*n* = 106, blue bars) as the denominator.

To detect the presence of the intact (functional) and deleted *norV* variants, all 303 O157 clinical isolates were examined. A 470 bp product representing the presence of a non-functional *norV* containing a 204 bp deletion was found in 197 (65.0%) strains. By contrast, a 675 bp product representing the intact and functional version of *norV* was found in the remaining 106 (35.0%) strains. Differences in the distribution of the intact *norV* were observed across the O157 clades previously classified using SNP genotyping. [10]. Among the predominant O157 clades examined, the non-functional *norV* containing the deletion was detected only in strains belonging to clades 1–3, while the intact *norV* was detected in all strains representing clades 6–9 (Figure 1). Among those 197 strains containing the 204 bp deletion, most (83.2%) belonged to clade 2, the predominant lineage examined in this subset of clinical strains. Comparatively, most of the strains containing the intact *norV* belonged to clades 8 and 7.

The non-random distribution of the two *norV* variants across the SNP-based phylogeny [10] was also linked to the distribution of *stx* subtypes. The 303 strains evaluated had seven different *stx* profiles that varied by clade and *norV* type (Table 2). Strains with the intact, functional *norV* possessed all seven *stx* variants, though some were more common than others. Strains with an intact *norV*, for instance, were significantly more likely to have either *stx2* alone or with *stx2c* compared to the remaining strains with an intact *norV* as well as those with a deleted version (Fisher’s exact test *p* ≤ 0.0001 and *p* ≤ 0.0001, respectively). Of the 81 strains containing *stx2* only (*n* = 41) or *stx2* with *stx2c* (*n* = 40), most (*n* = 63) belonged to clade 8. By contrast, strains with the deleted version of *norV* were significantly more likely to possess both *stx1* and *stx2* relative to all other strains (Fisher’s exact test *p* ≤ 0.0001). Among the 186 strains with *stx1* and *stx2*, most belonged to clades 2 (*n* = 152) and 3 (*n* = 32).

### 3.2. Impact of NO on stx2 Expression in the Clade 8 Outbreak Strain

**Figure 2:**
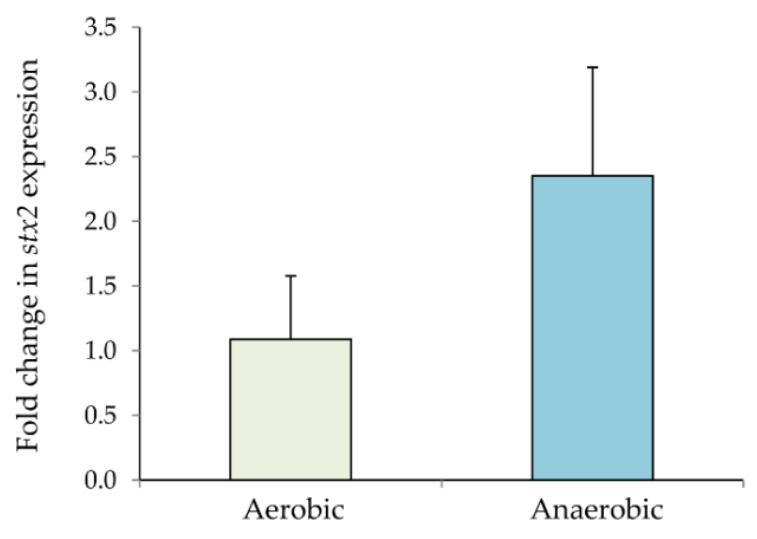
Fold change in *stx2* gene expression in the *E. coli* O157:H7 outbreak strain, TW14359, in the presence of NO in aerobic and anaerobic conditions after 24 h of growth. The *stx2* transcripts were normalized to the 16 S rRNA transcripts and are shown relative to the level of transcription in the absence of NO. The means of at least three independent experiments are shown by growth condition and the bars represent the standard deviation of the means. A fold-change ≥2 was considered biologically significant.

To determine whether strain TW14359 had higher *stx2* expression in response to NO, the strain was grown in LB with and without the NOR4 nitric oxide donor. In aerobic conditions, the *stx2* expression levels were not significantly altered in the presence or absence of NO after 6 h of growth (data not shown). The same was true in anaerobic conditions after 6 h. Increasing the incubation time, however, was associated with an increase in *stx2* expression but only in anaerobic conditions. Specifically, after 24 h of anaerobic growth, *stx2* expression was 2.2-fold higher in the presence of NO relative to the absence of NO (Figure 2). No significant difference was observed for aerobic conditions after 24 h of growth, indicating that NO activates *stx2* expression in the clade 8 spinach outbreak strain only in anaerobic conditions.

### 3.3. Role of norV in stx2 Expression in the Clade 8 Outbreak Strain across Growth Conditions

**Figure 3:**
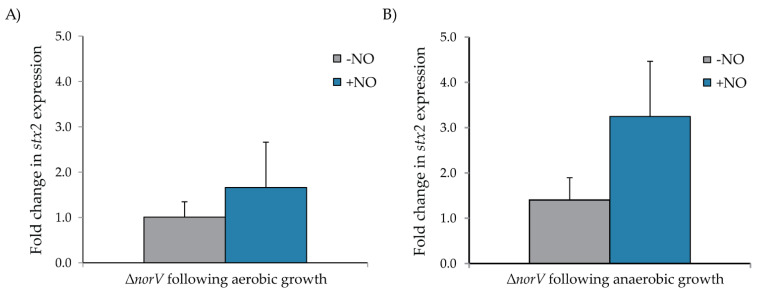
Fold change in *stx2* gene expression in (**A**) aerobic and (**B**) anaerobic conditions with and without nitric oxide (NO) in the null Δ*norV* mutant strain relative to the wild-type (WT) TW14359 O157:H7 strain. The *stx2* transcripts in each condition were normalized to the amount of 16S transcripts and are shown relative to the level of transcription in the WT for each growth condition. The boxes represent the means of at least three independent experiments and the bars are the standard deviation of the means. A fold-change ≥2 was considered biologically significant.

Since *norV* contributes to NO detoxification and an intact *norV* was present in all 66 clade 8 strains examined, we constructed an isogenic non-polar null *norV* mutant (Δ*norV*) in the wild-type (WT) spinach outbreak strain TW14359. *stx2* expression was quantified in Δ*norV* relative to the WT strain in the presence and absence of NO in both aerobic and anaerobic conditions following 24 h of growth. Under aerobic conditions, *stx2* transcription levels were similar between the WT and Δ*norV* in the presence and absence of NO (Figure 3A). As compared to the WT strain, *stx2* transcription levels were 1.0 ± 0.3 in the absence of NO and 1.7 ± 0.6 with NO. This finding suggests that *norV* does not affect *stx2* expression in aerobic conditions regardless of NO availability. By contrast, growth in anaerobic conditions for 24 h showed a difference in *stx2* expression in the presence of NO (Figure 3B). Specifically, the mutant Δ*norV* strain had a 3.2-fold increase in *stx2* expression as compared to the WT strain when NO was present. The WT and Δ*norV* revealed similar transcript levels of *stx2* in the absence of NO, suggesting that *norV* inhibits *stx2* expression solely in the presence of NO.

Next, we complemented the *norV* deletion in trans by expressing the WT *norV* on the pCR2.1-TOPO plasmid in the Δ*norV* strain (Δ*norV*::*norV*_WT_). *norV* expression in the complemented Δ*norV::norV*_WT_ strain was confirmed by qPCR, though the level of *norV* transcription was higher in the complemented strain than the WT. We then compared *stx2* expression levels in the WT, Δ*norV*, and Δ*norV::norV*_WT_ strains as well as the Δ*norV* strain containing the empty vector (Δ*norV::vector*) using the same conditions. Increased expression in *stx2* was observed in the Δ*norV* strain relative to the WT (data not shown*),* yet a similar increase was observed in both the Δ*norV::norV*_WT_ and Δ*norV::vector* strains.

### 3.4. Role of norV in Uptake and Survival of the Clade 8 Outbreak Strain within Macrophages

**Figure 4:**
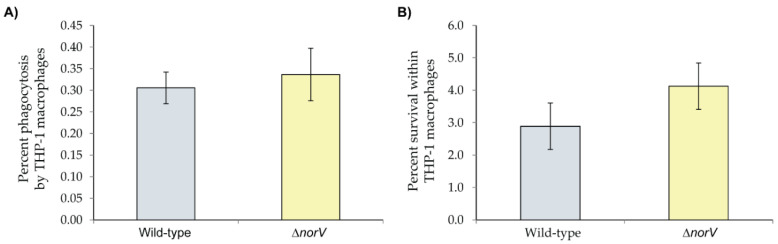
(**A**) Phagocytic uptake and (**B**) intracellular survival of the *E. coli* O157:H7 wild-type (WT) TW14359 outbreak strain relative to the isogenic Δ*norV* strain in THP-1 macrophages. Uptake was examined 1 h post-infection and survival was determined after 24 h. Boxes represent the means of three independent replicates and the bars show the standard deviation of the means. No significant differences were observed between strains for either phagocytic uptake or intracellular survival using a Student’s *t*-test.

To determine how *norV* impacts phagocytic uptake and intracellular survival of TW14359, we infected human THP-1-derived macrophages with the WT and isogenic Δ*norV* strains. At 1 h post infection, no difference in phagocytic uptake was observed between the WT and Δ*norV* strains (Figure 4A). Specifically, 0.31% ± 0.04 of the total WT cells per well and 0.34% ± 0.06 of Δ*norV* cells were taken up by the THP-1 macrophages after 1 h. Enhanced survival within THP-1 macrophages, however, was observed for the Δ*norV* strain 24 h post infection relative to the WT (Figure 4B). Nonetheless, this difference was not statistically significant (Student’s *t*-test *p* = 0.10). After normalizing to initial uptake levels, the percent survival of the WT and Δ*norV* strains within macrophages was 2.9% ± 0.7 and 4.1% ± 0.7, respectively.

An examination of *stx2* expression in the WT versus Δ*norV* strain following infection of THP-1 macrophages showed no significant difference in expression levels 1 h post infection (Student’s *t*-test *p* = 0.12). On average, the Δ*norV* strain had a 1.4-fold decrease in *stx2* expression relative to the WT. Expression of *stx2* at 24 h post infection could not be quantified for either strain as the number of transcripts was below the level of detection.

## 4. Discussion

EHEC O157 strains belonging to the clade 8 lineage were shown to be more commonly isolated from HUS cases than other lineages [10] and have an enhanced ability to adhere to epithelial cells and express *stx2* and other key virulence genes [11]. These findings suggest that clade 8 strains may be more virulent than strains representing other O157 lineages. Indeed, a prior study demonstrated enhanced virulence of the clade 8 spinach outbreak strain, K3995, relative to other O157:H7 strains in both rabbits and mice, which was due to increased Stx2 production [28]. This 2006 multistate spinach outbreak also contributed to a higher rate of HUS than has been observed for other O157:H7 outbreaks [13]. Additionally, a genome sequence comparison between the clade 8 spinach outbreak strain, TW14359, and two O157:H7 outbreak strains that caused low HUS case rates, found *norV* to be unique to the clade 8 strain [14]. Our evaluation of 303 O157 isolates, which represented the major phylogenic lineages previously defined by SNP genotyping [10], showed that 65% possess a 204 bp deletion in *norV* and that all of these strains belong to clades 2 and 3. The genome sequence of the EDL933 outbreak strain representing clade 3 also shows a deletion in *norV* [16] as does the clade 1 strain, RIMD 050995, linked to a 1996 outbreak in Sakai Japan [17]. Comparatively, the intact, functional version of *norV* was found in 35% of the 303 O157 strains evaluated herein and was restricted to clades 6–9.

Additional support for the distribution of the intact and deleted versions of *norV* was previously observed in different O157 strain collections. The initial screen of 100 O157 strains recovered from multiple sources detected the 204 bp deletion in 58% of strains and a correlation with *stx1* [14]. In our analysis, however, the deletion was most common among strains possessing both *stx1* and *stx2* representing clades 2 and 3, which indicates variation across the strain collections. This variation is not surprising given that Stx-encoding bacteriophages can integrate within multiple sites in different *E. coli* genomes [29]; hence, geographic variation could be due to the prevalence of circulating bacteriophages in certain locations. Consistent with our data, another screen of 107 distinct O157 strains confirmed the *norV* deletion to be restricted to clades 1–3, while the intact *norV* was correlated with clades 6–8 [30]. These findings indicate that *norV* functionality is linked to the genetic background of a given O157 strain. Additionally, the intact *norV* was detected in a subset of 34 EHEC strains representing 10 non-O157 serotypes [31], suggesting a more widespread distribution. The association observed between clade 8 strains, which all contained an intact *norV*, and the presence of *stx2* alone or with *stx2c* illustrates the importance of the O157 genetic background on virulence. Indeed, other strains with both an intact or deleted version of *norV* possess these *stx* subtypes either alone or in combination with *stx1*, suggesting that other factors also contribute to enhanced virulence in clade 8 strains. Examining *norV* function and its relationship with virulence is therefore warranted in a larger collection of clade 8 strains.

In anaerobic conditions, we demonstrated that NO induces *stx2* expression in the clade 8 spinach outbreak strain, TW14359, resulting in a > 2-fold increase of *stx2* transcription compared to growth in the absence of NO. In aerobic conditions, however, similar *stx2* transcription levels were observed in the presence and absence of NO. As the primary habitat for EHEC during infection is in the oxygen restricted intestine, it can encounter stressful conditions that may include NO produced by macrophages or endothelial and epithelial cells [32]. Our data showing increased *stx2* expression in TW14359 in anaerobic conditions, which mimic the gut environment, differ from responses observed in other O157 strains. For instance, in the clade 3 O157:H7 EDL933 outbreak strain, NO contributed to a decrease in Stx2 production by repressing the SOS response via the nitrite-sensitive repressor (NsrR) that senses NO [33]. NO was also shown to reduce expression of LEE-associated virulence genes, resulting in decreased adherence to epithelial cells in vitro [34]. These results, however, differ from those generated in another study using the same strain, EDL933, which found low NO concentrations induce production of Stx2 in anaerobic conditions [35]. The same study showed that a higher concentration of NO was needed for Stx1 production as well as Stx2 production in aerobic conditions, highlighting the complexity of NO signaling in EHEC O157.

Since NorV was previously shown to reduce NO, we sought to investigate the relationship between NorV and *stx2* expression in the clade 8 TW14359 strain. Notably, the Δ*norV* null mutant showed increased expression of *stx2* relative to the wild-type (WT) strain, indicating that NorV inhibits *stx2* expression in this strain. Expression of *norV* in trans on a plasmid in the Δ*norV* strain, however, failed to decrease *stx2* expression to levels similar to those observed for the WT strain. This failure to complement the WT phenotype is likely due to the use of ampicillin during the growth of the complemented strain since the pCR2.1-TOPO plasmid has an ampicillin resistance gene for selection. Multiple antibiotics, including ampicillin, have previously been shown to induce *stx* expression [36]. Contrary to our findings, Shimizu et al. [18] showed that by replacing the deleted *norV* copy found in the clade 3 EDL933 strain with an intact *norV* copy, *stx2* expression was increased in the presence of NO. This finding suggests that NorV induces the expression of *stx2* in EDL933. Moreover, another study showed that deleting *norVW* in the O157:H7 strain 620 was linked to a reduction in Stx production and persistence in the murine gut during infection; however, deletion of *norVW* in an O91:H21 strain (B2F1) had no impact on Stx production or persistence [31]. The reasons behind these phenotypic differences are not completely understood, although they are likely due to a variation in gene regulation and expression across O157 strains.

As part of the innate immune response to eliminate bacterial pathogens, NO is produced by the inducible NO synthase (iNOS) pathway within macrophages [37], which subsequently induces the bacterial SOS response. Therefore, we examined the role of *norV* in the survival of the O157:H7 strain TW14359 within THP-1 derived macrophages. Compared to the WT strain, the Δ*norV* mutant was not impaired in its ability to survive within macrophages. This finding suggests that NorV is not required for NO detoxification by strain TW14359 to enhance survival inside macrophages and that other factors may be necessary. Our preliminary finding that *norV* expression did not differ in TW14359 in the presence or absence of NO (*n* = 2; data not shown) provides further support for this hypothesis. This finding, however, requires confirmation and an additional set of clade 8 strains should be evaluated to better define strain-specific responses.

In addition to NorV, EHEC was shown to possess *hmp* and *hcp* encoding a flavohemoglobin (Hmp) [38] and high affinity NO reductase (Hcp) [39], respectively, which act independently to lower the toxicity of NO in different conditions [40]. Therefore, it is likely that either Hmp or Hcp assist with NO detoxification in TW14359 to promote survival inside macrophages and that one or both products could compensate for the loss of NorV activity. Indeed, NorV and Hmp were previously found to work synergistically during anaerobic growth [40]. Our results are consistent with those from another study demonstrating that survival of a *norV* mutant in the non-pathogenic *E. coli* strain MG1655 was not impaired in J774.2 mouse derived macrophages compared to the wild-type strain [41]. Similarly, no significant differences were observed in the survival of a *Salmonella enterica* Typhimurium strain within murine macrophages relative to the isogenic mutant lacking *norV* [42]. Contrary to our results, however, the number of surviving bacterial cells within murine macrophages following infection with the EHEC O157 K15 strain containing an intact *norV* was greater than the number following infection with a *norV* deficient mutant [18]. While this finding suggests that *norV* confers a protective advantage in resisting killing within macrophages for strain K15, the discrepant results are most likely due to the use of different O157 strains. Unlike TW14359, which belongs to clade 8 and contains an intact *norV*, strain K15 was found to represent clade 7 [30], the lineage linked to less severe symptoms in our prior study [10], despite having an intact *norV*. There is also the possibility that the RAW264.7 mouse derived macrophage cells, which were used to examine the survival of the EHEC K15 strain after 10 h [18], may differ from the human derived THP-1 cells used herein. Indeed, a prior study observed key differences in protein expression profiles between mouse-derived RAW264.7 macrophages and THP-1 cells [43].

In summary, our study shows that NO induces the expression of *stx2* in anaerobic conditions in the clade 8 spinach outbreak strain, TW14359, thereby increasing its pathogenicity. With that in mind, selected inhibition of NO production in the host could become the focus of extensive pharmaceutical research in the future. We also demonstrated that NorV inhibits *stx2* expression in TW14359, which is the opposite of what we might expect when considering the enhanced virulence of clade 8 strains. While inhibiting Stx production is important for ensuring bacterial survival, a prior study showed that varying NO concentrations can induce different Stx production pathways [35], further highlighting the complex relationship between NO signaling and Stx regulation. These data are relevant since the level of Stx inhibition likely varies across conditions and environments encountered in the host. As we have shown that O157 strains can have variable levels of *stx* expression [11,44,45], it is clear that strain-specific factors can also impact transcriptional regulation. Given the importance of strain-specific differences, a more comprehensive investigation into how *norV* impacts *stx* expression in other clade 8 strain backgrounds is therefore warranted. Lastly, in contrary to data generated in another study [18], we showed that *norV* is not required for the survival of TW14359 within THP-1 macrophages. This finding further suggests that the mechanisms for surviving in the presence of nitrosative stress, particularly following phagocytic uptake, vary across strains and that these mechanisms are complex and may require multiple factors that differ depending on the environment.

## 5. Conclusions

Collectively, our investigation of NorV in a clade 8 strain from a 2006 multistate outbreak linked to spinach consumption suggests that it plays a role in the inhibition of Stx2 production. The specific mechanism of inhibition, however, remains unclear and requires further study.

## Figures and Tables

**Table 1:** Oligonucleotide primers developed for use in this study.

Primer	Sequence (5′-3′)	Target	Amplicon Size
norv698_F	ATAACCCGACGCAAATTGT	*norV*	675 bp (intact) 470 bp (deletion)
norv1373_R	TATCCGGGACTTCACTCCA		
			
norV-del1	GCAATTAGCAAGACATCTTTTTAGAACACGCTGAATAAATTGAGGTTGCTGTGTAGGCTGGAGCTGCTTC	Kanamycin marker flanked by 50 bp of *norV*	1577 bp
norV-del2	CACCAGTTGGCGGGCGGCGAAGCCCGAACCAATGATCACAATGCCGTTGCCATATGAATATCCTCCTTAG		
			
norV-del3	GATATTCGCCAGCACATCAA	Δ*norV*	1868 bp (TW14359) 1905 bp (Δ*norV*)
norV-del4	AAACTGCTCGGCAAATTCAC		
			
norvcompl_F	AGATATTGTCATATCGACCATTGGA	*norV* + flanking region and *norV* promoter	1620 bp
norvcompl_R	AATGATCACAATGCCGTTGC		

**Table 2:** Distribution of *stx* profiles by clade and *norV* type among 303 Shiga toxin-producing *Escherichia coli* O157 clinical isolates.

Clade	*norV* †	*stx1*	*stx1*, *stx2*	*stx1*, *stx2*, *stx2c*	*stx1*, *stx2c*	*stx2*	*stx2*, *stx2c*	*stx2c*	Total *
2	Deletion	2 (1.2)	152 (92.7)	−	−	10 (6.1)	−	−	164
3	Deletion	1 (3.0)	32 (97.0)	−	−	−	−	−	33
6	Intact	−	−	−	−	1 (12.5)	1 (12.5)	6 (75.0)	8
7	Intact	1 (3.5)	−	1 (3.5)	7 (24.1)	−	6 (20.7)	14 (48.3)	29
8	Intact	−	1 (1.5)	1 (1.5)	−	30 (45.5)	33 (50.0)	1 (1.5)	66
9	Intact	−	1 (33.3)	−	2 (66.7)	−	−	−	3

* Percentages were calculated using the total number of isolates per clade as the denominator. † Differences in the frequencies of *stx* profiles by *norV* type were detected using the likelihood chi-square test; the Fisher’s exact test was used for sample sizes less than five.

## Data Availability

All data supporting the results can be found within the manuscript.
